# Initial or salvage treatment with apatinib shows promise against radioiodine-refractory differentiated thyroid carcinoma

**DOI:** 10.1530/ETJ-21-0065

**Published:** 2022-01-21

**Authors:** Xian Qiu, Lin Cheng, Ri Sa, Hao Fu, Yuchen Jin, Libo Chen

**Affiliations:** 1 Department of Nuclear Medicine, Shanghai Jiao Tong University Affiliated Sixth People’s Hospital, Shanghai, China; 2 Department of Nuclear Medicine, The First Hospital of Jilin University, Changchun, China; 3 Department of Nuclear Medicine & Minnan PET Center, The First Affiliated Hospital of Xiamen University, Xiamen, China; 4 Human Oncology and Pathogenesis Program, Memorial Sloan-Kettering Cancer Center, New York, New York, USA

**Keywords:** differentiated thyroid cancer, tyrosine kinase inhibitor, salvage treatment, apatinib, sorafenib

## Abstract

**Objective:**

Sorafenib and lenvatinib have been recommended as standard tyrosine kinase inhibitors (TKIs) for progressive radioiodine-refractory differentiated thyroid carcinoma (RR-DTC). However, their efficacy remains limited with unresolved drug resistance. Therefore, we conceived this open-label study based on real-world evidence to investigate the efficacy and safety of apatinib in patients with progressive RR-DTC.

**Methods:**

Off-label use of apatinib as either initial treatment or salvage treatment for sorafenib resistance was investigated. The primary endpoint was progression-free survival (PFS) and the secondary endpoints included objective response rate (ORR), overall survival (OS), and safety.

**Results:**

For all 28 enrolled patients, the median PFS was 15.1 months, with an ORR of 69.6%. The median OS was not reached at the data cut-off. In detail, the median PFS of 17.3 months and the ORR of 75% were determined in patients with TKI-naive RR-DTC (initial treatment group, *n*  = 14). And, in patients with first-line sorafenib-resistant RR-DTC (salvage treatment group, *n*  = 14), a median PFS of 12.0 months was reached, with an ORR of 45.5%. In the salvage treatment group, the median OS from the start of apatinib administration was 20.6 months, reaching 89.1 months from sorafenib treatment initiation. Adverse events at grade 3 or higher occurred in 64.3% of all subjects treated with apatinib.

**Conclusions:**

This study demonstrated that apatinib shows promise against RR-DTC with tolerable toxicity, representing a novel initial treatment for progressive RR-DTC and effective salvage treatment for RR-DTC resistant to sorafenib.

## Introduction

Thyroid cancer is responsible for 586,000 cases worldwide, ranking the ninth place for incidence in 2020, with differentiated thyroid cancer (DTC) accounting for over 90% (1). For DTC at an early stage, the optimal prognosis is commonly achieved because of its indolent nature and adequate management strategies, including surgery, radioiodine (^131^I) treatment, and levothyroxine therapy (2). However, persistent/recurrent or metastatic DTC, which is refractory to ^131^I therapy and/or tumor progression despite thyroid-stimulating hormone (TSH) suppression, has become the main cause of disease-specific death with a 10-year survival rate as low as 10% (3). The therapeutic efficacy of conventional strategies for cancers, including chemotherapy and external beam radiation therapy, remains limited in these challenging settings.

In recent years, tyrosine kinase inhibitors (TKIs) such as sorafenib and lenvatinib have highlighted new therapeutic options for the treatment of radioiodine-refractory DTC (RR-DTC), as clinical benefits have been achieved in patients with locally advanced or metastatic progressive RR-DTC, with significantly increased progression-free survival (PFS) (4, 5). However, limited response rates and drug resistance have also been reported. An objective response rate (ORR) of 12.2% in the sorafenib treatment cohort was reported by the phase 3 clinical trial DECISION (4). Disease progression was observed in approximately 7–30% of patients despite sorafenib or lenvatinib administration, and secondary drug resistance was observed in more than half of the patients in the first 2 years (5, 6, 7, 8).

To date, only a few clinical trial data on salvage therapy for patients with first-line TKI resistance have been available. A quarter of patients in the SELECT trial were treated with second-line lenvatinib, yielding an ORR of 62.1% and a PFS of 15.1 months, while overall survival (OS) remained unknown (5). Besides, a disappointing ORR of 15% was reported in the phase 3 clinical trial of cabozantinib as salvage therapy for patients with first-line TKI resistance (9). Nevertheless, real-world data on salvage therapy of RR-DTC resistant to first-line TKIs remain unavailable.

Apatinib, an orally active TKI that potently targets vascular endothelial growth factor receptor 2 (VEGFR2) and platelet-derived growth factor receptor, has been approved for the treatment of advanced gastric cancer refractory to two or more lines of prior chemotherapy (10). More recently, an overwhelming objective response to apatinib in progressive RR-DTC was reported, with optimistic PFS (22.2 months in apatinib group vs 4.47 months in placebo group) and OS (not reached in apatinib group vs 29.9 months in placebo group). However, data on salvage therapy using apatinib in this entity are lacking, hampering sufficient exhibition of its efficacy and safety profile in the treatment of RR-DTC.

Therefore, we conceived this open-label study based on real-world evidence to evaluate response, PFS, OS, and adverse effects in a cohort of consecutive RR-DTC patients treated with apatinib either as first line or as second line after the failure of sorafenib.

## Patients and methods

### Study populations

Patients with progressive RR-DTC lesions were eligible for enrollment. As described by our team, the diagnosis of RR-DTC relied on one of the following: (i) the foci did not concentrate ^131^I; (ii) despite previous evidence of ^131^I concentration, the foci lost the ability to concentrate ^131^I; (iii) concentration presented in some foci but not in others; and (iv) disease progressed within 1 year after ^131^I therapy (12). All patients with RR-DTC presented evidence of disease progression according to Response Evaluation Criteria in Solid Tumors (RECIST) version 1.1 within 12 months before apatinib treatment despite receiving sufficient levothyroxine to maintain a serum TSH level under 0.1 mIU/L (13). For therapeutic efficacy evaluation, patients were classified into two clinical situations: TKI-naive patients with RR-DTC diagnosed after ^131^I therapy (initial treatment group) and patients with RR-DTC who had failed to first-line sorafenib treatment (salvage treatment group). Patients with concurrent cancers beyond DTC were excluded. Premenopausal women were required to undergo negative pregnancy tests, and all patients with childbearing potential were required to use contraception. Patients were treated independently of the Eastern Cooperative Oncology Group performance status (ECOG PS), systematic chemotherapy, and life expectancy.

The ethics board of Shanghai Jiao Tong University Affiliated Sixth People’s Hospital approved the protocol prior to the study. All subjects provided written informed consent for participation in the study.

### Study design

The objective of this study was to reveal the full landscape in patients with RR-DTC following the off-label use of apatinib by providing data on the response, PFS, OS, and safety.

Apatinib was orally administered at an initial dose of 250 mg twice a day until discontinued owing to disease progression, uncontrollable side effects, death, or at the patient’s request. Drug interruption, dose reduction (250 mg or 125 mg per day), and drug withdrawal were permitted when grade 3 or higher adverse events (AEs) occurred. Screening evaluations, including medical history, demography, review of prior treatment, physical examination, baseline imaging, and laboratory evaluations, were completed within 1 week before the start of apatinib administration.

Following treatment initiation, patients were observed at intervals of 2–4 weeks. At each visit, a complimentary medical history inquiry and physical examination were performed, and the levels of serum TSH, thyroglobulin (Tg), and anti-Tg antibody (TgAb) were measured by electrochemiluminescence immunoassay on a Cobas analyzer (Roche Diagnostics Gmbh, Roche Ltd.). Safety and tolerability were monitored simultaneously. The severity of AEs was graded according to the National Cancer Institute Common Terminology Criteria for Adverse Events (version 4.03) (14). The radiologic response was assessed every 2–3 months.

### Therapeutic efficacy evaluation

Radiographic assessments were performed by competent radiologists using CT, MRI, or PET combined with diagnostic CT. The radiologic response was defined according to RECIST 1.1 as follows: complete response, disappearance of all target lesions. Any pathologic lymph nodes (target or non-target) should demonstrate a reduction in short axis to <10 mm; partial response (PR), ≥30% decrease in the sum of diameter of target lesions; progressive disease, ≥20% increase in the sum of diameter of target lesions or appearance of new lesions; stable disease (SD), neither sufficient shrinkage to qualify for PR nor sufficient increase to be eligible for PD (13). Patients without target lesions who were excluded from the radiologic response analysis were included in the survival analysis.

The primary endpoint of this study was PFS, defined as the time from the initiation of apatinib treatment to disease progression or death, whichever occurred first. Secondary study endpoints included safety, ORR, disease control rate (DCR), time to response (TTR), duration of response (DoR), and OS. ORR was defined as the proportion of patients who had a CR or PR as the best response, and DCR was defined as the proportion of patients who had a CR, PR, or SD as the best response. TTR was defined as the time interval between the first dose of apatinib and the initial objective response (CR or PR). DoR was defined as the time from first documented evidence of CR or PR until the time of first documented disease progression or death (any cause), whichever occurred first. OS was defined as the time from the first dose of apatinib to death from any cause.

The biochemical evaluation was utilized as a supplement to the structural assessment. ∆Tg%, change rate in Tg level, was defined as follows: (Tg_baseline_ − Tg_min_)/Tg_baseline_ × 100%. Tg_baseline_ and Tg_min_ referred to the serum Tg level immediately before apatinib treatment and the lowest serum Tg level during the treatment, respectively. ∆Tg% < −25.0% and ∆Tg% ≥ 25.0% indicated biochemical progression and biochemical response, respectively. Biochemical stabilization was defined as −25.0% ≤ ∆Tg% <25.0% (15). Patients were excluded from the analysis of biochemical evaluation when serum TgAb > 100 IU/mL (16).

### Statistical analyses

All analyses were conducted using SPSS software version 20.0 (IBM Corp.). Descriptive statistics included median and minimum and maximum continuous factors and scores. In the case of categorical variables, numbers and percentages were compared using the chi-square test. The cumulative probabilities of PFS and OS were calculated using Kaplan–Meier survival estimates. A log-rank test was used to test for differences between time-to-event curves. Two-tailed probabilities were reported, and a *P* -value <0.05 was used to define nominal statistical significance.

## Results

### Baseline characteristics

In total, 28 patients were enrolled with ratio of males to females of nearly 1.8:1. The median age was 57.3 years (range, 24.2–78.3 years). An ECOG PS of >2 was observed in five (17.9%) patients. More than 85% of patients were pathologically diagnosed with papillary thyroid cancer. Except for four (14.3%) patients with locally advanced disease, lung (71.4%) and bone (35.7%) were the most frequently involved organs by DTC metastasis. The median time interval between DTC diagnosis and apatinib administration was 9.4 years (range, 0.8–25.2 years). The median follow-up duration from apatinib initiation to treatment cessation or death was 20.3 months (range, 2.2–53.2 months). The baseline clinicopathological characteristics of all the subjects are summarized in Table 1 with regard to clinical scenarios: all enrolled patients with progressive RR-DTC (*n*  = 28), TKI-naive patients with RR-DTC diagnosed after ^131^I therapy (initial treatment group, *n*  = 14), and patients with RR-DTC who had failed to first-line sorafenib treatment (salvage treatment group, *n*  = 14).

**Table 1:** Baseline characteristics of differentiated thyroid carcinoma patients treated with apatinib.

Patient characteristics	All patients, *n* (%)	Initial treatment group, *n* (%)	Salvage therapy group, *n* (%)
Gender			
Female	12 (42.9)	4 (28.6)	7 (50.0)
Male	16 (57.1)	10 (71.4)	7 (50.0)
Age			
≥55 years	15 (53.6)	7 (50.0)	8 (57.1)
<55 years	13 (46.4)	7 (50.0)	6 (42.9)
ECOG PS			
0–2	23 (82.1)	13 (92.9)	10 (71.4)
3–4	5 (17.9)	1 (7.1)	4 (28.6)
Histology			
PTC	24 (85.7)	12 (85.7)	12 (85.7)
FTC	4 (14.6)	2 (14.3)	2 (14.3)
Initial AJCC stage			
I–II	21 (75.0)	10 (71.4)	11 (78.6)
III–IV	7 (25.0)	4 (28.6)	3 (21.4)
Tumor extent			
Locally advanced	4 (14.3)	2 (14.3)	2 (14.3)
Lymph-node metastasis	21 (75.0)	10 (71.4)	11 (78.6)
Lung metastasis	21 (75.0)	8 (57.1)	13 (92.9)
Bone metastasis	12 (42.9)	8 (57.1)	4 (28.6)
Brain metastasis	2 (7.1)	1 (7.1)	1 (7.1)
Pleura metastasis	3 (10.1)	2 (14.3)	1 (7.1)
Paranephric metastasis	3 (10.1)	2 (14.3)	1 (7.1)
Muscle metastasis	1 (3.6)	0 (0.0)	1 (7.1)
Previous treatment			
^131^I therapy	28 (100.0)	14 (100.0)	14 (100.0)
EBRT	2 (7.1)	2 (14.3)	0 (0.0)
Sorafenib	14 (50.0)	0 (0.0)	14 (100.0)
^131^I-avidity			
Presence	14 (59.1)	3 (21.4)	10 (71.4)
Absence	14 (40.9)	11 (78.6)	4 (28.6)
Mean Tg ng/mL^a^, ± s.d.	2050.5 ± 3816	2132 ± 3075	1948 ± 3075

^a^Patient from salvage treatment group was excluded from the biochemical assessment due to TgAb > 100 IU/mL.

DTC, differentiated thyroid cancer; EBRT, external beam radiation therapy; ECOG PS, Eastern Cooperative Oncology Group performance status; FTC, follicular thyroid cancer; PTC, papillary thyroid cancer; Tg, thyroglobulin.

### Response to apatinib

**Figure 1:**
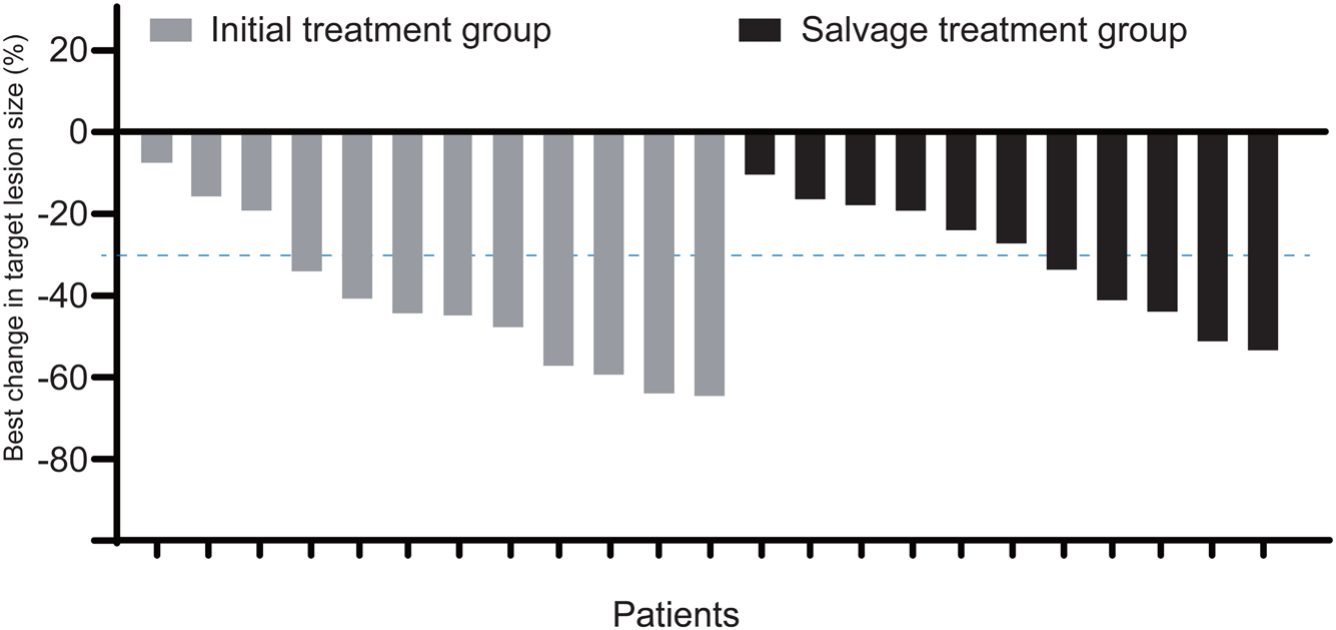
Best changes in the sum of the largest diameter of target lesions from baseline. Initial treatment group, tyrosine kinase inhibitor-naive patients with progressive radioiodine-refractory DTC (RR-DTC), *n*  = 12; Salvage treatment group, patients with first-line sorafenib-resistant RR-DTC, *n*  = 11.

After excluding 3 patients who died before the first radiologic assessment and 2 patients without a target lesion, the remaining 23 patients (12 in the initial treatment group and 11 in the salvage treatment group) were eligible for the assessment of radiologic response. No CR was observed. PRs were achieved in nine and five patients in initial treatment group and salvage treatment group, respectively, yielding an ORR of 75.0% (95% CI, 0.47–1.08) and 45.5% (95% CI, 0.10–0.81), respectively. Furthermore, the sum of the diameter of target lesions decreased in all the patients, manifesting a DCR of 100% (Fig. 1).

The median TTRs in the initial treatment group and salvage treatment group were 5.7 months (range, 2.8–10.2 months) and 6.4 months (range, 1.8–12.8 months), respectively. Subsequently, the median DoRs were estimated as 12.1 months (range, 5.0–25.0 months) and 8.2 months (range, 0.5–11.8 months), respectively.

Owing to serum TgAb > 100 IU/mL, one patient was excluded from the biochemical assessment using the change in Tg level. Of the remaining 27 patients, the mean serum Tg level decreased significantly from 2050.5 ± 3816 to 880.3 ± 1463 ng/mL, reaching the nadir within approximately 11.9 weeks after treatment initiation, yielding overall biochemical response and stabilization rates of 81.5% and 18.5%, respectively. In detail, 11 (78.6%) and 10 (76.9%) patients attained biochemical response in initial treatment group and salvage treatment group, respectively. None of the patients demonstrated biochemical progression.

### Survival analyses

**Figure 2:**
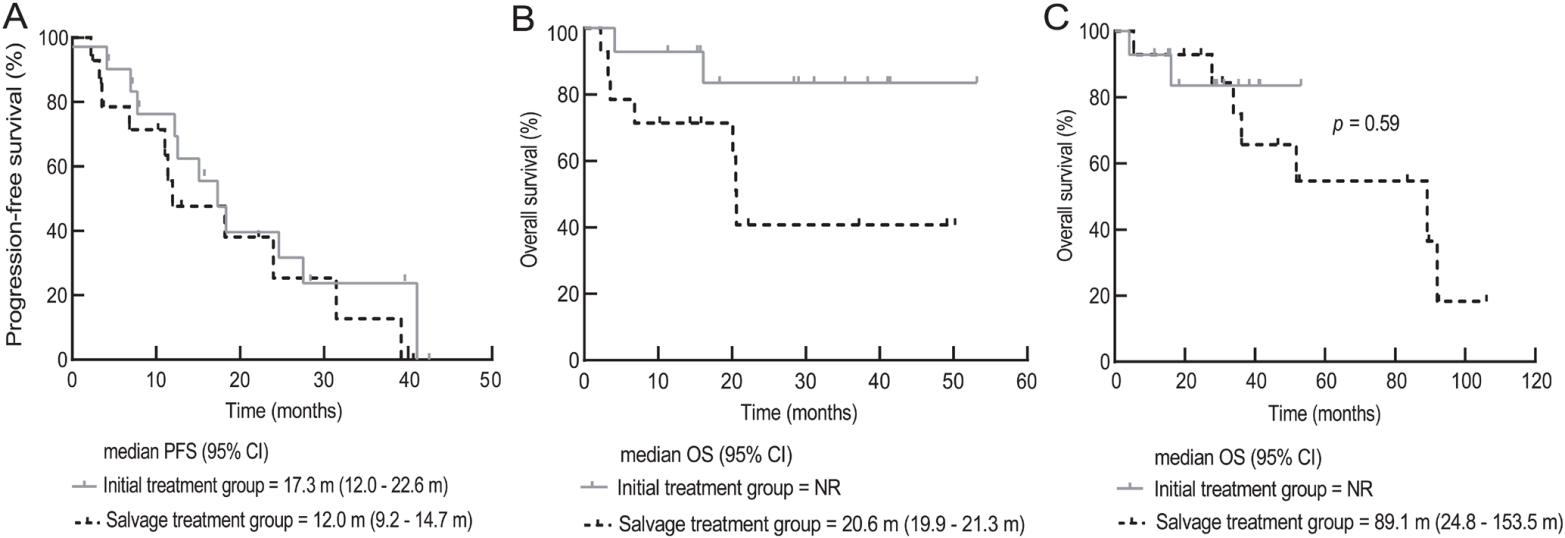
Survival benefit in patients with radioiodine-refractory differentiated thyroid cancer (RR-DTC) treated with apatinib. Progression-free survival (PFS) (A) and overall survival (OS) (B) in the full analysis sets. The OS curves of initial treatment group and salvage treatment group from the first dose of tyrosine kinase inhibitor (C). Initial treatment group, tyrosine kinase inhibitor-naive patients with progressive RR-DTC, *n*  = 14; Salvage treatment group, patients with first-line sorafenib-resistant RR-DTC, *n*  = 14. NR, not reached.

Median PFS were 15.1 months (95% CI, 7.3–22.9 months), 17.3 months (95% CI, 12.0–22.6), and 12.0 months (95% CI, 9.2–14.7) in all the enrolled patients, initial treatment group, and salvage treatment group, respectively (Fig. 2A).

Median OS in either all patients or initial treatment group was not observed at the data cut-off (Fig. 2B). In the salvage treatment group, the median OS from the initiation of apatinib administration was 20.6 months (95% CI, 19.9–21.3 months) (Fig. 2B), while the overall median OS from the initiation of sorafenib as initial TKI therapy through the administration of apatinib as salvage treatment was 89.1 months. Kaplan–Meier survival estimate of the entire median OS starting from the first dose of TKI showed no significant difference between initial treatment group and salvage treatment group (*P*  = 0.59) (Fig. 2C).

### Adverse events

AEs were reported in 100% of patients receiving apatinib treatment (Table 2). AEs in grades ≥ 3 occurred in 18 (64.3%) of the 28 enrolled patients. The most frequent AEs were proteinuria (67.9%), followed by hypertension (53.6%), hand-foot syndrome (46.4%), diarrhea (39.3%), increased alanine aminotransferase and/or aspartate aminotransferase (28.6%), oral mucositis (28.6%), fatigue (28.6%), hypocalcemia (17.9%), and headache (14.3%). The mean sustainable daily dose of apatinib was 299 ± 106 mg. Drug interruptions, dose reductions, and drug withdrawals owing to AEs occurred in 5 (17.9%), 19 (67.9%), and 4 (14.2%) patients, respectively.

**Table 2:** Treatment-emergent adverse event profile according to common terminology criteria for adverse events (version 4.03) (*n* = 28).

Adverse events	All grades, *n* (%)	Grade ≥ 3, *n* (%)
Any treatment-related adverse effect	28 (100.0)	18 (64.3)
Proteinuria	19 (67.9)	3 (10.1)
Hypertension	15 (53.6)	8 (28.6)
Hand–foot syndrome	13 (46.4)	2 (7.1)
Diarrhea	11 (39.3)	5 (17.9)
Nausea	2 (7.1)	0 (0.0)
Increased aminotransferase	8 (28.6)	2 (7.1)
Oral mucositis	8 (28.6)	1 (3.6)
Hypocalcemia	5 (17.9)	4 (14.3)
Headache	4 (14.3)	0 (0.0)
Fatigue	8 (28.6)	0 (0.0)
Weight loss	4 (14.3)	2 (7.1)
Skin ulceration	1 (3.6)	1 (3.6)
Voice change	1 (3.6)	0 (0.0)
Neutrophil count decreased	1 (3.6)	0 (0.0)
Platelet count decreased	1 (3.6)	1 (3.6)
Hypokalemia	2 (7.1)	1 (3.6)

## Discussion

The present open-label study comprehensively investigated the efficacy and safety of apatinib based on real-world data in the treatment of patients with progressive RR-DTC, including patients with no history of TKI administration and patients who failed first-line sorafenib treatment. Recently, phase 2 and 3 trials of apatinib illustrated sustainable efficacy and safety for patients with progressive RR-DTC (, 17). However, they might not fully reveal the clinical settings in real world compared with the current study, in which subjects with a broader scope of ECOG PS (0–4) and those without target lesions were enrolled. Although the sample size in each group was relatively small owing to the scarcity of patients presenting the above lethal conditions, the current single-arm study comprehensively revealed the landscape of therapeutic outcomes in these currently challenging malignancies treated with apatinib, warranting randomized, controlled clinical trials to optimize drug selection for progressive RR-DTC patients in the era of targeted therapy of cancer. Notably, all target lesions shrank with favorable prognosis in both clinical scenarios, suggesting that apatinib at an initial dose of 250 mg twice daily could be a promising strategy not only as initial TKI treatment for progressive RR-DTC patients but also as salvage treatment when prior TKI fails.

**Figure 3:**
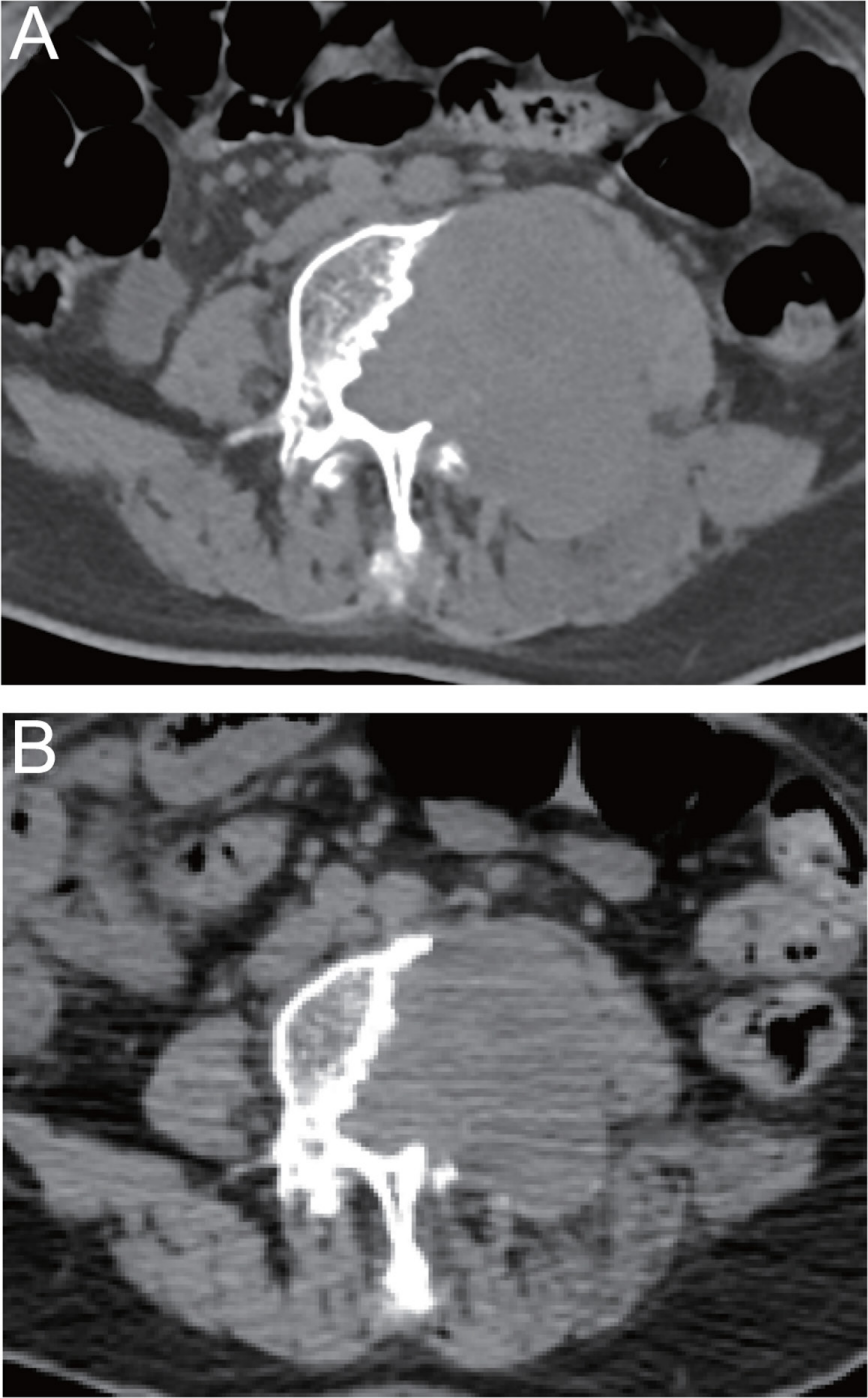
Regression of a bone metastatic radioiodine-refractory follicular thyroid cancer lesion in a 74-year-old male patient. Despite ^131^I-avidity demonstrated by post-therapeutic ^131^I scan, the metastatic lesion in fourth lumbar vertebra is enlarged one month after ^131^I administration (A), resulting in an inability to walk. Five months after the initiation of apatinib treatment, the patient could walk again, with the CT scan showing a 15.0% decrease in the largest diameter of the mass from baseline (B).

Despite the approval of sorafenib and lenvatinib in patients with progressive RR-DTC, we still explored the novel TKI, apatinib, as initial therapy for this malignancy owing to the overwhelming efficacy previously recognized by our team and others (12, 18, 17), with the aim to develop more efficacious options for patients with progressive RR-DTC. As expected, the median PFS of patients with progressive RR-DTC who initially received apatinib as TKI therapy was 17.3 months, which seems comparable with those obtained by sorafenib (9–24 months) or lenvatinib (18.7 months) treatment (5, 7, 15, 19, 20). Moreover, an ORR of 75% for initial treatment group is also more favorable than those observed with sorafenib (12.2–31%) and lenvatinib (64.8%) (19, 4, 5, 20). As previously reported by our team and other research groups, bone metastases from DTC remain commonly resistant to ^131^I or sorafenib alone (7, 21), and apatinib alone or in combination with ^131^I may be a reasonable attempt to achieve more favorable outcomes (Fig. 3). Furthermore, the favorable efficacy of apatinib as an initial TKI treatment in patients with progressive RR-DTC may warrant head-to-head comparisons between TKIs against RR-DTC to optimize drug selection.

**Figure 4:**
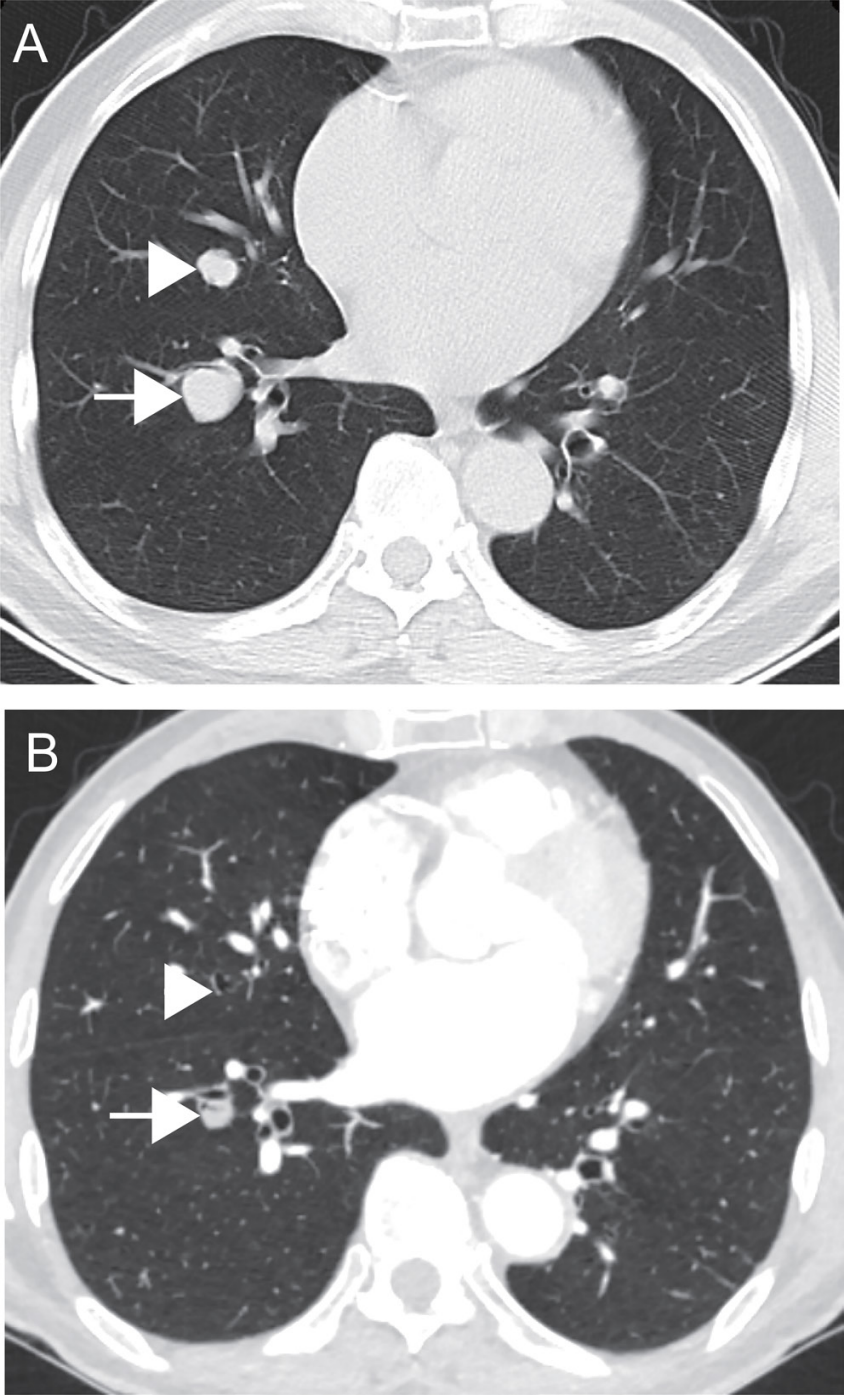
Notable shrinkage of a sorafenib-resistant radioiodine-refractory lung metastatic papillary thyroid cancer in a 62-year-old male patient treated with apatinib. (A) CT planar scan before apatinib treatment; (B) CT planar scan 5 months after apatinib initiation, showing a 67% decrease in the sum of the target lesion. Simultaneously, the serum thyroglobulin level dramatically declined from 938 to 38 ng/mL.

To date, salvage therapy including sunitinib, pazopanib, cabozantinib, and lenvatinib have been evaluated for sorafenib-resistant RR-DTC, with a limited additional median PFS of 4–15.1 months and total OS of 58 months (5, 22). Notably, our patients who had previously failed first-line sorafenib could benefit from apatinib salvage treatment, as an additional median PFS of 12.0 months and total median OS of 89 months were achieved. Furthermore, the median OS from the time point of disease progression after first-line sorafenib treatment was 20.6 months, which is much longer than that of 4.9 months in patients who did not receive salvage treatment (8). And, the ORR of apatinib was a lit bit lower than that in the SELECT trial, which may be due to the potential bias derived from small sample size, decreased administered doses, and higher ECOG performance status but far better than that of cabozantinib, indicating the considerable efficacy of apatinib as salvage therapy (5, 9). To the best of our knowledge, this is the first study utilizing apatinib as a salvage agent in the treatment of RR-DTC resistant to first-line sorafenib, revealing a potential role in these intractable entities (Fig. 4). Interestingly, comparable median OS could be achieved between the group using apatinib as initial TKI therapy and the group together with salvage treatment for sorafenib resistance using apatinib. Concerning its substantial effect on tumor shrinking, we speculate that apatinib as a first-line TKI may be more suitable for patients who need impending relief of tumor-related symptoms and signs, while sorafenib may be preferred as first-line TKI for asymptomatic patients and those with subtle clinical manifestations considering its more favorable tolerance (7). Additionally, the median OS has not yet been achieved in patients treated with apatinib as initial TKI therapy, indicating the necessity of longer follow-ups and comparisons between a variety of sequential treatment schemes.

Moreover, a relatively higher incidence of AEs has been reported in the 750 mg daily apatinib regime than the 500 mg daily regime (23). Considering the convincing response to 500 mg per day in DTC and anaplastic thyroid cancer previously verified by our team (7, 12), we maintained the regimen of 250 mg apatinib twice a day as an initial dosage for over 4 years. Notably, the present study demonstrated that excellent PFS in each group could be achieved by a median maintenance dose of nearly 300 mg per day. As the incidence of AEs at any grade and grade 3 or higher in the 500 mg daily regime reached 100 and 64.3%, respectively, and dose reduction due to AEs occurred in more than half of patients, we suggest that precaution, intensive care, and frequent examinations be considered to monitor patients and guide dose modulations.

## Conclusions

In summary, the current study revealed that apatinib treatment at a starting dose of 250 mg twice daily demonstrated potent and durable therapeutic efficacy, representing a promising initial treatment for TKI-naive progressive RR-DTC, as well as effective salvage treatment for first-line sorafenib-resistant RR-DTC. Prolonged analyses with larger sample sizes and investigations on the alleviation of apatinib-induced AEs are needed.

## Declaration of interest

The authors declare that there is no conflict of interest that could be perceived as prejudicing the impartiality of the research reported.

## Funding

This study was sponsored by the National Natural Science Foundation of China http://dx.doi.org/10.13039/501100001809 Grant (No. 81671711).

## Author contribution statement

All authors participated in the study’s conceptualization; data collections were by all the authors; Xian Qiu and Lin Cheng participated in data analysis; Xian Qiu, Lin Cheng, and Libo Chen participated in writing the original draft; All authors edited and reviewed draft.
